# Minyoo Matata – The Vicious Worm – A *Taenia solium* Computer-Based Health-Education Tool – in Swahili

**DOI:** 10.1016/j.pt.2017.05.012

**Published:** 2017-10

**Authors:** Chiara Trevisan, Eric M. Fèvre, Maurice Owiny, Isaac Ngere, Maria Vang Johansen

**Affiliations:** 1 Department of Biomedical Sciences, Institute of Tropical Medicine, Nationalestraat 155, 2000 Antwerp, Belgium; 2 Institute of Infection and Global Health, University of Liverpool, Leahurst Campus, Chester High Road, Neston, CH64 7TE, UK; 3 International Livestock Research Institute, Old Naivasha Road, P.O. Box 30709-00100, Nairobi, Kenya; 4 Kenya Field Epidemiology and Laboratory Training Programme, P.O. Box 22313-00100, Nairobi, Kenya; 5 Department of Veterinary and Animal Sciences, University of Copenhagen, Dyrlægevej 100, 1870 Frederiksberg C, Denmark

## Abstract

Lack of knowledge is one of the main risk factors for the spread of the zoonotic parasite *Taenia solium.* The computer-based health-education tool ‘The Vicious Worm’ was developed to create awareness and provide evidence-based health education as a specific measure in control strategies. To increase the reach of the tool, a new version in Swahili was developed and can now be downloaded for free from http://theviciousworm.sites.ku.dk.

## Improved Knowledge: A Key Strategy for the Control of *Taenia solium*

*Taenia solium* taeniosis/cysticercosis is a zoonotic parasitic disease that contributes to substantial public health and economic consequences across the globe [1]. It is endemic in areas where pigs are raised in free-ranging systems, where sanitation facilities are inadequate, where personal and meat hygiene are deficient, and where information and knowledge on the parasite are lacking. A significant part of the control of this pathogen rests on educating residents of endemic communities about the risks factors, transmission routes, diagnostic tools, and transmission prevention and control. Improved knowledge through health education was suggested by the World Health Organization (WHO) as one of the five key strategies for the control of *T. solium* taeniosis/cysticercosis (http://www.who.int/neglected_diseases/NTD_RoadMap_2012_Fullversion.pdf).

## What Is ‘The Vicious Worm’?

‘The Vicious Worm’ is an open-access digital-based health-education tool for *T. solium* taeniosis/cysticercosis. The English version of the programme was developed in 2014 by the University of Copenhagen as part of an EU-7th framework funded programme Integrated Control of Neglected Zoonoses in Africa (ICONZ) with the hope of having evidence-based health education included as a specific control measure in any control strategy [2]. The tool was designed to create awareness of *T. solium* taeniosis/cysticercosis at all levels, from policy makers to school children in rural communities. The computer programme provides information on transmission, diagnosis, treatment, risk factors, prevention, and control of *T. solium* taeniosis/cysticercosis, and targets a range of stakeholders across different sectors and disciplines. The educational materials included in ‘The Vicious Worm’ are illustrated short stories, videos, scientific texts, and policy and information sheets displayed using an interactive map and separating issues for a generalised ‘village’, ‘town’, and ‘city’ audience. Simple information on the parasite for lay people and children can be found at ‘village level'; technical information for health and agriculture professionals and students can be found at ‘town level'; and information for national and international policy makers can be found at ‘city level’.

## How to Increase the Impact of the Health-Education Tool

‘The Vicious Worm’ was tested in Tanzania on agriculture and veterinary officers and health workers and had a positive impact among these stakeholders, as they significantly improved their knowledge after using the programme [3].

As all age groups are susceptible to the disease, in order to be effective, health education should target the population at all socioeconomic and age levels [4]. In East Africa, where cysticercosis is a problem across several countries [5], Swahili is the most commonly spoken language (with more than 50 million speakers) [6]. To increase the use and impact of this health-education tool in East African communities where English is not always spoken, a Swahili version of the full tool was created.

## Minyoo Matata: The Vicious Worm Now Also in Swahili

**Figure 1:**
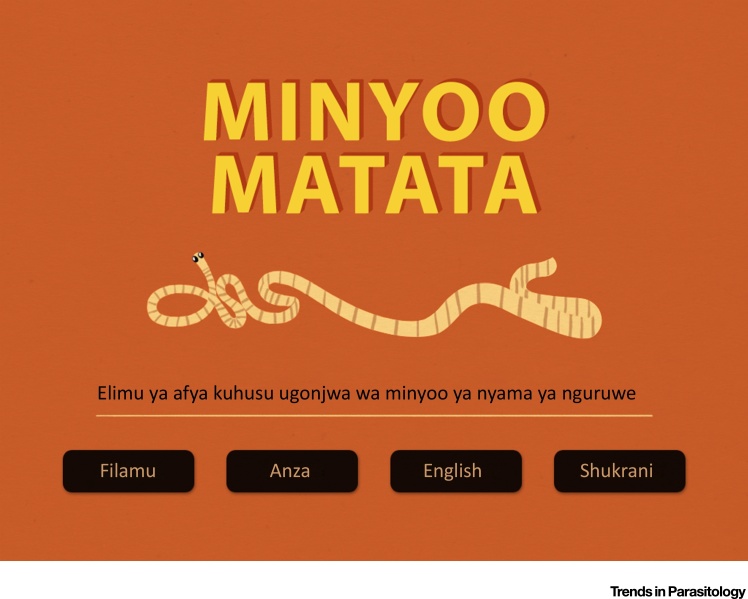
Title Page of Minyoo Matata – The Vicious Worm – A *Taenia solium* Taeniosis/Cysticercosis Health-Education Tool – in Swahili.

The new version of ‘The Vicious Worm’ was produced in collaboration with the original developers at the University of Copenhagen and researchers at the University of Liverpool and the International Livestock Research Institute in Kenya. For the new version, the educational material of the English version was systematically translated and back-translated to ensure accuracy, formatted, and implemented in a new version. The beta version of the ‘Minyoo Matata’ – The Vicious Worm – A *Taenia solium* Taeniosis/Cysticercosis Health-Education Tool – in Swahili is now available and can be downloaded for free through the homepage http://theviciousworm.sites.ku.dk. This new version of the programme allows the switching of languages between English and Swahili (Figure 1). English and Swahili versions of the programme are also available as an Android app for android tablets and smart-phones and can be downloaded for free through the Android app store.

## The Way Forward

Control of *T. solium* taeniosis/cysticercosis is in large part dependent on behavioural changes in pig-keeping and at-risk communities, and ‘The Vicious Worm’ tool is effective in providing the information that leads to those behavioural changes. Previous targeting has, however, focussed on adults, and further work is required to assess the effectiveness of the tool in children and, indeed, the onward transmission of information and health messages from adults to children and vice versa. We expect that the impacts of health education using the tool in both adults and children will be more effective in Swahili, which will broaden the relevance of the tool to the large group of Swahili speakers in East and Central Africa.

In East Africa, well over 60% of mobile phones now sold are data-enabled smart-phones, and many internet kiosks or cyber-cafes sell short-term internet access through smart-phones and computers (http://www.ca.go.ke/images/downloads/STATISTICS/Sector%20%20Statistics%20Report%20Q1%202015-16.pdf). In addition, computers are increasingly available in state-operated schools. Throughout Kenya, approximately 15 000 000 school children have access to information and communication technologies (e.g., http://cfsk.org/); our hope is that the tool will be widely accessible and may also be useful to teachers in comparing Swahili and English versions.

While the programme will be targeted to those areas where this endemic disease is a significant problem, translating the tool to Swahili will theoretically make it accessible and appropriate for more than 50 million Swahili speakers in East Africa [6]. Better knowledge at all levels will help to achieve the WHO's goal of eliminating this public and animal health problem (http://www.who.int/neglected_diseases/NTD_RoadMap_2012_Fullversion.pdf), a goal which is fully aligned with a number of programmes on disease surveillance and control currently taking place in endemic countries across the globe.
